# The burdens of poverty during the COVID-19 pandemic

**DOI:** 10.3389/fsoc.2022.995318

**Published:** 2022-11-24

**Authors:** Julia Petersen, Nora Hettich, Rieke Baumkötter, Philipp S. Wild, Norbert Pfeiffer, Thomas Münzel, Jochem König, Karl J. Lackner, Manfred E. Beutel

**Affiliations:** ^1^Department of Psychosomatic Medicine and Psychotherapy, University Medical Center Mainz, Mainz, Germany; ^2^Preventive Cardiology and Preventive Medicine, Center for Cardiology, University Medical Center Mainz, Mainz, Germany; ^3^German Center for Cardiovascular Research (DZHK), Partner Site Rhine Main, Mainz, Germany; ^4^Department of Ophthalmology, University Medical Center Mainz, Mainz, Germany; ^5^Center for Cardiology, University Medical Center Mainz, Mainz, Germany; ^6^Division of Pediatric Epidemiology, Institute of Medical Biostatistics, Epidemiology and Informatics, University Medical Center Mainz, Mainz, Germany; ^7^Institute of Clinical Chemistry and Laboratory Medicine, University Medical Center Mainz, Mainz, Germany

**Keywords:** SARS-CoV-2, COVID-19 pandemic, poverty, economic burden, psychological stress

## Abstract

**Background:**

Individuals living at-risk-of-poverty have an increased risk of poor mental health. The pandemic and its societal impacts might have negative effects especially on this group widening the gap between rich and poor and also exacerbate gender gaps, which in turn might impact social cohesion.

**Aim:**

The objective of this longitudinal study was to determine if people living at-risk-of-poverty were more vulnerable to economic and psychosocial impacts of the pandemic and showed poorer mental health. Moreover, gender differences were analyzed.

**Method:**

We drew data from a sample of *N* = 10,250 respondents of two time points (T1 starting from October 2020, T2 starting from March 2021) of the Gutenberg COVID-19 Study. We tested for differences between people living at-risk-of-poverty and more affluent respondents regarding economic impacts, psychosocial stressors, as well as depressiveness, anxiety and loneliness, by comparing mean and distributional differences. To test for significant discrepancy, we opted for chi-square- and *t*-tests.

**Results:**

The analysis sample compromised *N* = 8,100 individuals of which 4,2% could be classified as living at-risk-of-poverty. 23% of respondents living at-risk-of-poverty had a decrease in income since the beginning of the pandemic–twice as many as those not living at-risk-of-poverty, who reported more often an increase in income. Less affluent individuals reported a decrease in working hours, while more affluent people reported an increase. Between our survey time points, we found a significant decrease in these economic impacts. Gender differences for economic changes were only found for more affluent women who worked more hours with no change in income. Less affluent respondents were more impacted by psychosocial stressors, depressiveness, anxiety, and loneliness. Gender differences were found particularly with regard to care responsibilities.

**Discussion:**

Our results indicate a widening in the gap between the rich and the poor at the beginning of the pandemic. Gender differences concerning economic changes affect more affluent women, but women in both income groups are more burdened by care responsibilities, which might indicate a heightened resurgence of gender role in times of crisis. This increase in inequality might have impacted social cohesion.

## Introduction

Although the coronavirus disease 2019 (COVID-19) pandemic constitutes a health-related crisis, it rapidly became clear that this could also dovetail with a social and economic crisis, particularly for already vulnerable individuals. Poverty is an important risk factor for poor physical and mental health. Even before the COVID-19 pandemic, people with a low income had a higher vulnerability to suffer from chronic diseases and mental health problems (Aue et al., 2016).

As the measures taken by governments around the world to combat the spread of the COVID-19 pandemic changed daily life and work tremendously, numerous jobs were lost and social welfare institutions suspended their help temporarily (Brodeur et al., 2021). The probability to become a person at-risk-of-poverty [60 % of the median net equivalized income of all households in a country (Eurostat, n.d)] grew during this time (Brodeur et al., 2021). However, previous studies mainly focused on social inequity (education, income, areas of living) as risk factor to get infected with the virus. As for mental health impacts during the pandemic, longitudinal studies using samples of the general population found mainly slight increases in depressiveness, anxiety, and loneliness during the pandemic (Peters et al., 2020; Pierce et al., 2020; Kivi et al., 2021; Kwong et al., 2021). Reviews and meta-analyses confirmed small but significant negative effects on mental health symptoms of anxiety and depression (Kunzler et al., 2021; Prati and Mancini, 2021). Effects for loneliness, general distress, negative affect, and suicide risk were not significant (Prati and Mancini, 2021; Ernst et al., 2022). Some studies identified lower socioeconomic status, unemployment, being female, pre-existing mental conditions, chronic diseases, increased exposure to infection, and being younger as risk factors for poor mental health (Daly et al., 2020; Peters et al., 2020; Santabárbara et al., 2020; Breslau et al., 2021; Fancourt et al., 2021; Kunzler et al., 2021; Kwong et al., 2021; Niedzwiedz et al., 2021; Benatov et al., 2022; Bonati et al., 2022; Saeed et al., 2022). Low education or income, female gender, young age, having a long-term medical condition, or a history of mental illness were identified as risk factors for loneliness during the pandemic (Bu et al., 2020; Varga et al., 2021; Jaspal and Breakwell, 2022). Most of those risk factors are also known as potential predictors for poverty, indicating an association between the two pandemic impacts.

Already before the pandemic, associations between inequality or poverty, social cohesion, and mental health have been found. We understand social cohesion to consist of three main dimensions: social relations, identification, and orientation toward a common good (Schiefer and van der Noll, 2017). Kawachi and Kennedy (1997) argued that an increase in income inequality leads to an increase in the concentration of poverty and affluence, which in turn might lead to population health impacts due to deteriorating social cohesion. They stated that this might be because inequality negatively impacts crime rates, economic productivity, and the functioning of a representative democracy and thus society and social cohesion themselves. Furthermore, Fone et al. (2007) provided evidence that poor mental health outcomes were associated with neighborhood income deprivation and low social cohesion, indicating a joint effect. In a later study, Fone et al. (2014) also found evidence for social cohesion acting as a mediator between living in deprived neighborhoods and change in mental health, significantly decreasing the effect of poverty on mental health if social cohesion is heightened. Hong et al. (2014) came to similar results for a Latino community. Furthermore, Chuang et al. (2013) found that respondents who lived in countries with higher social inclusion, social diversity, as well as social capital (which they argued to be aspects of social cohesion) were more likely to demonstrate good general health, with the effect of the social cohesion aspects outweighing even individual-level characteristics.

Scholars highlighted the association of social cohesion and mental health during the pandemic. Kim (2020) suggested that emotional and psychological stress due to uncertainty, not being able to participate in social life, and not being in control in times of a global pandemic might have reduced social cohesion, canceling out its protective nature. Silveira et al. (2022) also found that during the first lockdown the levels of social cohesion, as well as adaptive coping, decreased while psychological vulnerability increased, indicating a higher likelihood of negative mental health impact. Focusing on deprived and marginalized communities, studies also showed that social cohesion within these groups had been negatively impacted during the pandemic (Friedkin, 2004; Fone et al., 2007; Greene et al., 2015; Kim, 2020; Borkowska and Laurence, 2021; Silveira et al., 2022). Therefore, we suggest that growing economic inequality and a negative impact on mental health might also indicate a decline in social cohesion during the pandemic.

This study examined whether people at-risk-of-poverty were more likely to suffer from negative economic and employment impacts of the pandemic as well as from mental health burdens regarding depressiveness, anxiety, and loneliness. The aim of this paper was to investigate possible differences in depressiveness, anxiety, and loneliness between people living at risk of poverty and those above the threshold for poverty over the span of the pandemic. Potential stressors such as job loss, loss of working hours, and loss of income are considered. We also focused on the interaction with gender differences. Respondents of a large, population-based, prospective, observational single-center cohort study were examined. This paper contributes to the important issue of how the COVID-19 pandemic affects the mental health and social and economic situation of people at-risk- of-poverty and thereby might impact social cohesion in Germany.

The following questions were addressed:

1. Are persons at-risk-of-poverty more vulnerable to
  a. negative economic and employment impacts, and
  b. poor mental health during the COVID-19 pandemic?
2. Are there differences between women and men in less and more affluent individuals?
3. Are there differences between the two survey time points regarding the wealth and mental health gap?

## Methods

### Study design and sample

We draw our data from the Gutenberg COVID-19 Study (GCS), a population-representative, prospective cohort study. The study sample consists of *N* = 8,121 individuals of the Gutenberg Health Study [GHS, (Wild et al., 2012)] and *N* = 2,129 newly recruited individuals. The GHS is a large-scale population-based cohort study that focuses on a multitude of diseases, such as cardiovascular diseases, cancer, ophthalmological diseases, metabolic diseases, diseases of the immune system, and mental diseases and aims to improve the individual risk predication for diseases. After the outbreak of SARS-CoV-2, the respondents of the Gutenberg Health Study were invited to participate in the Gutenberg COVID-19 Study. The overall objective of the GCS is to comprehensively and systematically investigate the epidemiology of the COVID-19 pandemic in the population.

The recruitment process of the GHS started in 2007 in the target area of Mainz/Mainz-Bingen by drawing random samples from the resident's registration office. Women and men aged between 35 and 74 were invited to participate. The sample was stratified by gender, age, and place of residence (Mainz/Mainz-Bingen). Individuals who were mentally or physically unable to visit the study center as well as individuals with low proficiency in the German language were excluded from the study. For the GCS, 2129 additional respondents aged 25–44 years were additionally recruited. In total, the GCS cohort includes 10,250 individuals aged 25 to 88 years. In the context of the GCS, two visits at the study center took place, during which a computer-assisted personal interview and sequential sampling of biomaterial were performed. Questionnaires were sent prior to the visit at the study site. The first GCS data collection took place from October 2020 to April 2021 (T1), the second from March 2021 to June 2021 (T2). For the present study, we included respondents with available data at both measurement time points and household incomes. In addition, participants who are currently pursuing education were excluded from this study since it is difficult to compare full-time students with people who are already in the working sector. This left us with a sample of *N* = 8,100 individuals.

The requirements of Good Clinical Practice (GCP), Good Epidemiological Practice (GEP), and the ethical standards of the Declaration of Helsinki were considered during the study's design, implementation, and analysis. Furthermore, the Federal Data Protection Act's requirements were implemented. The Ethics Committee of the Rhineland-Palatinate Medical Association, as well as the Data Protection Officer of the Johannes Gutenberg University Hospital Mainz assessed all study-relevant documentation for the Gutenberg Health Study and the Gutenberg COVID-19 Study and gave a positive vote. The data protection commissioner of Rhineland-Palatinate approved the drawing of the sample *via* the citizens' registration offices.

### Measures

In order to measure mental health impacts, we used depressiveness, anxiety, loneliness, and psychosocial stress as indicators. For each time point, depressiveness was assessed using the self-administered Patient Health Questionnaire (PHQ-9) depression scale (Löwe et al., 2004). On a 4-point scale (0 = 'not at all' to 3 = 'nearly every day') respondents answered questions regarding their level of interest, eating habits, self-perception, capacity to concentrate and sleep, energy levels, feeling down or depressed, and thoughts of suicide. The items were summed up to create a composite score. Anxiety was measured using the GAD-2 questionnaire (Spitzer et al., 2006; Kroenke et al., 2007), a two-item screening instrument that asks respondents to score how much they have been impacted by uneasiness, anxiety, and the inability to stop or control their worrying on a scale of 0 ('not at all') to 3 ('nearly every day'). The two items were used as a sum score. The three-item loneliness scale (Hughes et al., 2004), shortened from the 20-item Revised UCLA Loneliness Scale [R-UCLA, (Russell et al., 1980)], was used to measure loneliness. Respondents were asked to rate on a scale ranging from 0 (“never”) to 4 (“always”) how often they lacked companionship, how often felt like left out, and how often they felt isolated from others. Furthermore, we included the psychosocial stress screening instrument PHQ-Stress (Gräfe et al., 2004). PHQ-Stress was measured by asking respondents to rate how much stressors such as worrying about health and looks, financial strain, and dreams about traumatic experiences has impacted them on a scale from 0 (“not bothered at all”) to 2 (“bothered a lot”).

We considered gender and being at-risk-of-poverty as main predictors. Being at-risk-of-poverty was estimated using relative poverty defined by the European Union Statistics on Income and Living Conditions [EU-SILC, (Eurostat, n.d)]. According to EU-SILC, a person is at risk of poverty if their net equivalized income is under 60% of the median net equivalized income of all households. Net equivalized income was calculated by dividing the total monthly net income of a household by a weighted household size. The first adult was weighed by a factor of 1,0, every additional household member over the age of 14 years of age was weighed by adding a factor of 0,5, and every child under the age of 14 years of age was weighed by adding a factor of 0,3 to the weighing scale. Since the median in 2019 was at 1,790€, we estimated a net equivalized of under 1,074€ to be the threshold of living at-risk-of-poverty.

Additionally, we inquired about a change in a person's income (no; yes, it has increased; yes, it has decreased; no answer) and about a change in a person's occupation (no; reduction of working hours; increase of working hours; job loss) in order to estimate the economic impact. At T1, respondents were asked about changes since the beginning of the pandemic. At T2, they were asked about changes since the last time they were surveyed. All measurement instruments were collected using a computer-assisted personal interview (CAPI).

### Statistical analysis

We first identified respondents who could be classified to live at-risk-of-poverty. We then performed a descriptive analysis to identify sociodemographic differences between people living at-risk-of-poverty and those who do not live at-risk-of-poverty. Secondly, we tested for further differences between the two groups and between the time points regarding economic impacts, psychosocial stressors, as well as depressiveness, anxiety and loneliness, by comparing mean and distributional differences. We opted for chi-square and *t*-tests in order to identify significant differences between the groups. A *p* < 0.05 indicated a significant discrepancy. All analyzing and testing was performed using R (Version 1.3.1093, packages: car, carData, dplyr, psych, sandwich, jtools, lm.beta).

## Results

### Sample characteristics

Within our sample (*N* = 8,100), 342 individuals were classified as individuals living at-risk-of-poverty according to the EU-SILC (see Table 1). In comparison to the rest of the participants, this population was significantly younger (more people between 25 and 34). In addition, less affluent individuals held lower education degrees, were significantly more often unemployed or worked irregularly, were more often single or lived apart from their partner, had more children under the age of 18 living in the same household, and had more frequently a migration background. We found no difference in COVID-infection between the two groups.

**Table 1:** Socio-demographic characteristics of respondents living and not living at-risk-of-poverty.

	**Sample**	**At-risk-of-poverty**	**Not at-risk-of-poverty**	
	**(*N* = 8,100)**	**(*N* = 342)**	**(*N* = 7.758)**	
	***N*** **(%)**	***N*** **(%)**	***N*** **(%)**	***p***
Gender				
Male	4,024 (49.7%)	154 (45.0%)	3,870 (49.9%)	0.089
Female	4,076 (50.3%)	188 (55.0%)	3,888 (50.1%)	
Age				
25–34	792 (9.8%)	52 (15.2%)	740 (9.5%)	**0.001**
35–44	1,221 (15.1%)	41 (12.0%)	1,180 (15.2%)	0.120
45–54	1,462 (18.0%)	61 (17.8%)	1,401 (18.1%)	0.974
55–64	1,868 (23.1%)	85 (24.9%)	1,783 (23.0%)	0.460
65–75	1,632 (20.1%)	63 (18.4%)	1,569 (20.2%)	0.456
75+	1,125 (13.9%)	40 (11.7%)	1,085 (14.0%)	0.263
Education				
No/ other degree	19 (0.3%)	3 (1.2%)	16 (0.3%)	**0.039**
Secondary general School	1,571 (23.8%)	109 (41.9%)	1,462 (23.1%)	**0.000**
Secondary School	1,676 (25.4%)	63 (24.2%)	1,613 (25.5%)	0.704
Academic secondary school	3,325 (50.5%)	85 (32.7%)	3,240 (51.2%)	**0.000**
Further education				
No/ other degree	225 (3.4%)	25 (9.6%)	200 (3.2%)	**0.000**
Vocational school	3,647 (55.3%)	181 (69.9%)	3,466 (54.7%)	**0.000**
University degree	2,719 (41.3%)	54 (20.8%)	2,665 (42.1%)	**0.000**
Employment status				
No current occupation	2,535 (33.2%)	127 (40.8%)	2,408 (32.8%)	**0.004**
Irregular	461 (6.0%)	56 (18.0%)	405 (5.5%)	**0.000**
Part-time	1,368 (17.9%)	64 (20.6%)	1,304 (17.8%)	0.236
Fulltime	3,280 (42.9%)	64 (20.6%)	3,216 (43.9%)	**0.000**
Partnership				
Single	1,223 (18.3%)	109 (39.1%)	1,114 (17.4%)	**0.000**
Partnership (living apart)	522 (7.8%)	58 (20.8%)	464 (7.2%)	**0.000**
Partnership (living together)	4,948 (73.9%)	112 (40.1%)	4,836 (75.4%)	**0.000**
Children under 18 in household (yes)	1,913 (23.6%)	85 (24.9%)	1,828 (23.6%)	0.628
Mean number of children under 18 in household	0.42 (0.94)	0.73 (2.12)	0.41 (0.85)	**0.000**
Migration background (yes)	1,703 (21.0%)	93 (27.4%)	1,610 (20.8%)	**0.004**
COVID-infection				
T1	293 (3.6%)	9 (2.6%)	284 (3.7%)	0.399
T2	404 (5.0%)	21 (6.1%)	383 (4.9%)	0.382

We used chi-square tests of independence to test for significant differences between the groups. Significant p-values in bold. T1 = COVID-Infection at survey time point 1. T2 = COVID-Infection at survey time point 2.

### Economic impacts

Individual economic and employment changes since the beginning of the COVID-19 pandemic for less and more affluent women and men are shown in Table 2. For changes in income, we found that individuals that were more affluent reported significantly more often no changes or higher income while less affluent persons reported significantly more often less income during the pandemic. This was found for both measurement times. Considering changes in employment, the analysis showed for the first time point (T1) that respondents living at-risk-of-poverty reported more frequently to have had no changes in working hours or worked fewer hours since the start of the pandemic. More affluent respondents, however, reported working more hours than before the pandemic. Only the difference that less affluent individuals reported fewer working hours during the pandemic remained significant at the second measurement point (T2). At T1, less affluent respondents reported significantly more often that they have received either short-time compensation or financial aid. At T2, less affluent respondents reported more often to have started a new job. When we looked at the changes over time, we found that the reported frequencies of respondents earning less income and working less significantly decreased for all respondents (see Appendix 1). Additionally, more affluent respondents stated less frequently that they worked more and had more income since the first survey time point.

**Table 2:** Changes in income and employment during the COVID-19 pandemic for men and women living and not living at-risk-of-poverty (*N* = 8,100).

	**At-risk-of-poverty**													
	**T1**							**T2**						
	**Sample**	**At-risk-of-poverty**			**Not at-risk-of-poverty**			**Sample**	**At-risk-of-poverty**			**Not at-risk-of-poverty**		
	**(*****N*** = **8,100)**	**(*****N*** = **342)**			**(*****N*** = **7,758)**			**(*****N*** = **8,100)**	**(*****N*** = **342)**			**(*****N*** = **7,758)**		
	**M (SD)**	**M (SD)**			**M (SD)**		***p***	**M (SD)**	**M (SD)**			**M (SD)**		***p***
**Change in personal income during pandemic**														
No	5,964 (74.7%)	233 (68.7%)			5,731 (75.0%)		**0.012**	6,135 (80.3%)	228 (73.6%)			5,907 (80.7%)		**0.003**
Yes, more	931 (11.7%)	19 (5.6%)			912 (11.9%)		**0.001**	678 (8.9%)	15 (4.8%)			663 (9.0%)		**0.014**
Yes, less	954 (11.9%)	78 (23.0%)			876 (11.5%)		**0.000**	601 (7.9%)	50 (16.1%)			551 (7.5%)		**0.000**
**Change in working hours/occupation**														
No	1,864 (23.0%)	102 (29.8%)			1,762 (22.7%)		**0.003**	3,484 (43.0%)	131 (38.3%)			3,353 (43.2%)		0.082
Yes, working less	553 (6.8%)	35 (10.2%)			518 (6.7%)		**0.015**	149 (1.8%)	14 (4.1%)			135 (1.7%)		**0.003**
Yes, working more	648 (8.0%)	17 (5.0%)			631 (8.1%)		**0.045**	449 (5.6%)	19 (5.6%)			430 (5.6%)		1.000
Yes, I got a new job	100 (1.2%)	4 (1.2%)			96 (1.2%)		1.000	80 (1.0%)	8 (2.3%)			72 (0.9%)		**0.021**
Yes, I lost my job	23 (0.3%)	3 (0.9%)			20 (0.3%)		0.112	10 (0.1%)	2 (0.6%)			8 (0.1%)		0.090
Yes, I received short-time compensation	123 (1.5%)	11 (3.2%)			112 (1.4%)		**0.016**	58 (0.7%)	1 (0.3%)			57 (0.7%)		0.534
Yes, I received financial aid	17 (0.2%)	3 (0.9%)			14 (0.2%)		**0.031**	8 (0.1%)	1 (0.3%)			7 (0.1%)		0.775
	**At-risk-of-poverty x gender**													
	**T1**							**T2**						
	**Sample**	**At-risk-of-poverty**			**Not at-risk-of-poverty**			**Sample**	**At-risk-of-poverty**			**Not at-risk-of-poverty**		
	**(*****N*** = **8,100)**	***Men***	***Women***		***Men***	***Women***		***N*** = **8,100**	***Men***	***Women***		***Men***	***Women***	
		**(*****N*** = **154)**	**(*****N*** = **188)**		**(*****N*** = **3,870)**	**(*****N*** = **3,888)**			**(*****N*** = **154)**	**(*****N*** = **188)**		**(*****N*** = **3,870)**	**(*****N*** = **3,888)**	
	**M (SD)**	**M (SD)**	**M (SD)**	***p***	**M (SD)**	**M (SD)**	***p***	**M (SD)**	**M (SD)**	**M (SD)**	***p***	**M (SD)**	**M (SD)**	***p***
**Change in personal income during pandemic**														
No	5,964 (74.7%)	106 (68.8%)	127 (68.7%)	1.000	2,859 (74.8%)	2,872 (75.2%)	0.721	6,135 (80.3%)	106 (76.3%)	122 (71.3%)	0.397	2,914 (79.6%)	2,993 (81.7%)	**0.019**
Yes, more	931 (11.7%)	10 (6.5%)	9 (4.9%)	0.680	476 (12.5%)	436 (11.4%)	0.171	678 (8.9%)	7 (5.0%)	8 (4.7%)	1.000	392 (10.7%)	271 (7.4%)	**0.000**
Yes, less	954 (11.9%)	35 (22.7%)	43 (23.2%)	1.000	441 (11.5%)	435 (11.4%)	0.864	601 (7.9%)	21 (15.1%)	29 (17.0%)	0.775	278 (7.6%)	273 (7.5%)	0.865
**Change in working hours/occupation**														
No	1,864 (23.0%)	54 (35.1%)	48 (25.5%)	0.072	927 (24.0%)	835 (21.5%)	**0.010**	3,484 (43.0%)	65 (42.2%)	66 (35.1%)	0.218	1,738 (44.9%)	1,615 (41.5%)	**0.003**
Yes, working less	553 (6.8%)	16 (10.4%)	19 (10.1%)	1.000	263 (6.8%)	255 (6.6%)	0.709	149 (1.8%)	6 (3.9%)	8 (4.3%)	1.000	61 (1.6%)	74 (1.9%)	0.310
Yes, working more	648 (8.0%)	4 (2.6%)	13 (6.9%)	0.115	268 (6.9%)	363 (9.3%)	**0.000**	449 (5.6%)	6 (3.9%)	13 (6.9%)	0.329	173 (4.5%)	257 (6.6%)	**0.000**
Yes, I got a new job	100 (1.2%)	1 (0.6%)	3 (1.6%)	0.761	39 (1.0%)	57 (1.5%)	0.085	80 (1.0%)	3 (2.0%)	5 (2.7%)	0.941	26 (0.7%)	46 (1.2%)	**0.026**
Yes, I lost my job	23 (0.3%)	1 (0.6%)	2 (1.1%)	1.000	9 (0.2%)	11 (0.3%)	0.831	10 (0.1%)	1 (0.7%)	1 (0.5%)	1.000	3 (0.0%)	5 (0.1%)	0.728
Yes, I received short-time compensation	123 (1.5%)	4 (2.6%)	7 (3.7%)	0.780	59 (1.5%)	53 (1.4%)	0.617	58 (0.7%)	0 (0.0%)	1 (0.5%)	1.000	30 (0.8%)	27 (0.7%)	0.777
Yes, I received financial aid	17 (0.2%)	0 (0.0%)	3 (1.6%)	0.321	5 (0.1%)	9 (0.2%)	0.427	8 (0.1%)	1 (0.7%)	0 (0.0%)	0.920	4 (0.1%)	3 (0.1%)	0.995

We used chi-square tests of independence to test for significant differences between the groups. Significant p-values in bold. Respondents who chose to not respond to the questions and data that was otherwise missing was excluded from this table, which is why the data of the columns might not add up to 100%.

When considering the interaction between risk-at-poverty and gender, we found that there were no significant differences in any economic impact between less affluent men and women. Between more affluent men and women, we found significant differences. Women reported to work more hours since the beginning of the pandemic at T1 and T2. At T2, more affluent men reported more often an increased income since the start of the pandemic while more affluent women reported more frequently no changes in income, but they have started more often a new job. As for changes between the time points, we, again, observed that, less respondents stated that they worked less and had a decreased income. Here, we also found that more affluent respondents, regardless of gender, reported significantly less that they worked more and had a higher income since the beginning of the pandemic.

### Psychosocial impacts

Differences in psychosocial stress (PHQ-stress) since the beginning of the COVID-19 pandemic between less and more affluent women and men are shown in Table 3. In general, people living at-risk-of-poverty reported a higher sum score of stress for both time points. On a single item level, financial, social, and traumatic concerns were higher for less affluent individuals for both time points. The only stressor that was more common amongst the more affluent respondents was a low or non-existing sexual desire at T1. Interestingly, at T1, less affluent individuals reported significantly more worrying about something bad that had happened recently, but this difference was no longer significant at T2. However, at T2, less affluent individuals reported significantly more worries about their health. When looking at significant differences between the time points, we found that while more affluent respondents reported significant increases in most items, less affluent respondents only reported increases for concerns for weight and looks as well as for lower libido and having no one to talk to (see Appendix 2).

**Table 3:** Stressors and burdens of men and women living and not living at-risk-of-poverty (*N* = 8,100).

	**At-risk-of-poverty**													
	**T1**							**T2**						
	**Sample**	**At-risk-of-poverty**			**Not at-risk-of-poverty**			**Sample**	**At-risk-of-poverty**			**Not at-risk-of-poverty**		
	**(*****N*** = **8,100)**	**(*****N*** = **342)**			**(*****N*** = **7,758)**			**(*****N*** = **8,100)**	**(*****N*** = **342)**			**(*****N*** = **7,758)**		
	**M (SD)**	**M (SD)**			**M (SD)**		***p***	**M (SD)**	**M (SD)**			**M (SD)**		***p***
Sum score PHQ stress	4.01 (3.17)	4.66 (3.47)			3.98 (3.15)		**0.000**	4.23 (3.34)	4.96 (3.58)			4.20 (3.33)		**0.000**
Concern about health	0.71 (0.65)	0.76 (0.70)			0.71 (0.65)		0.147	0.73 (0.67)	0.83 (0.68)			0.73 (0.67)		**0.008**
Concern about weight and looks	0.59 (0.67)	0.66 (0.67)			0.59 (0.67)		0.053	0.70 (0.69)	0.77 (0.71)			0.70 (0.69)		0.073
Low or no sexual desire or pleasure during intercourse	0.49 (0.65)	0.42 (0.62)			0.50 (0.65)		**0.042**	0.55 (0.67)	0.53 (0.66)			0.55 (0.67)		0.540
Problems with spouse or (life) partner	0.38 (0.59)	0.44 (0.65)			0.38 (0.59)		0.075	0.41 (0.61)	0.44 (0.60)			0.41 (0.61)		0.385
Burden of caring for children, parents or other family members	0.45 (0.66)	0.46 (0.65)			0.45 (0.66)		0.804	0.43 (0.65)	0.46 (0.67)			0.43 (0.65)		0.343
Stress at work or in school	0.59 (0.73)	0.54 (0.72)			0.59 (0.73)		0.181	0.58 (0.72)	0.54 (0.72)			0.58 (0.72)		0.293
Financial issues or concerns	0.23 (0.49)	0.66 (0.72)			0.21 (0.47)		**0.000**	0.22 (0.48)	0.65 (0.71)			0.20 (0.46)		**0.000**
Having no one to talk to about issues	0.24 (0.49)	0.34 (0.55)			0.24 (0.48)		**0.000**	0.36 (0.59)	0.47 (0.66)			0.36 (0.59)		**0.001**
Something bad that happened recently	0.26 (0.57)	0.36 (0.65)			0.26 (0.56)		**0.002**	0.26 (0.57)	0.32 (0.61)			0.26 (0.57)		0.055
Thoughts or dreams about bad events^a^	0.23 (0.50)	0.34 (0.61)			0.22 (0.50)		**0.000**	0.21 (0.48)	0.33 (0.57)			0.20 (0.48)		**0.000**
	**At-risk-of-poverty x gender**													
	**T1**							**T2**						
	**Sample**	**At-risk-of-poverty**			**Not at-risk-of-poverty**			**Sample**	**At-risk-of-poverty**			**Not at-risk-of-poverty**		
	**(*****N*** = **8,100)**	***Men***	***Women***		***Men***	***Women***		***N*** = **8,100**	***Men***	***Women***		***Men***	***Women***	
		**(*****N*** = **154)**	**(*****N*** = **188)**		**(*****N*** = **3,870)**	**(*****N*** = **3,888)**			**(*****N*** = **154)**	**(*****N*** = **188)**		**(*****N*** = **3,870)**	**(*****N*** = **3,888)**	
	**M (SD)**	**M (SD)**	**M (SD)**	***p***	**M (SD)**	**M (SD)**	***p***	**M (SD)**	**M (SD)**	**M (SD)**	***p***	**M (SD)**	**M (SD)**	***p***
Sum score PHQ stress	4.01 (3.17)	4.27 (3.54)	4.98 (3.40)	0.061	3.59 (2.96)	4.37 (3.29)	**0.000**	4.23 (3.34)	4.58 (3.47)	5.27 (3.64)	0.077	3.70 (3.17)	4.69 (3.40)	**0.000**
Concern about health	0.71 (0.65)	0.71 (0.69)	0.80 (0.71)	0.247	0.65 (0.63)	0.77 (0.66)	**0.000**	0.73 (0.67)	0.81 (0.70)	0.84 (0.66)	0.736	0.66 (0.65)	0.80 (0.68)	**0.000**
Concern about weight and looks	0.59 (0.67)	0.51 (0.63)	0.79 (0.68)	**0.000**	0.49 (0.62)	0.70 (0.70)	**0.000**	0.70 (0.69)	0.64 (0.69)	0.87 (0.71)	**0.003**	0.57 (0.64)	0.83 (0.71)	**0.000**
Low or no sexual desire or pleasure during intercourse	0.49 (0.65)	0.50 (0.64)	0.35 (0.59)	**0.030**	0.49 (0.64)	0.51 (0.66)	0.093	0.55 (0.67)	0.56 (0.66)	0.49 (0.66)	0.368	0.53 (0.66)	0.57 (0.68)	**0.004**
Problems with spouse or (life) partner	0.38 (0.59)	0.44 (0.65)	0.44 (0.65)	0.925	0.36 (0.57)	0.40 (0.61)	**0.004**	0.41 (0.61)	0.40 (0.58)	0.48 (0.62)	0.240	0.38 (0.59)	0.44 (0.63)	**0.000**
Burden of caring for children, parents or other family members	0.45 (0.66)	0.40 (0.65)	0.51 (0.65)	0.160	0.39 (0.61)	0.52 (0.70)	**0.000**	0.43 (0.65)	0.37 (0.61)	0.55 (0.71)	**0.017**	0.36 (0.60)	0.50 (0.70)	**0.000**
Stress at work or in school	0.59 (0.73)	0.42 (0.65)	0.62 (0.76)	**0.017**	0.54 (0.69)	0.65 (0.76)	**0.000**	0.58 (0.72)	0.47 (0.69)	0.59 (0.75)	0.164	0.52 (0.69)	0.65 (0.75)	**0.000**
Financial issues or concerns	0.23 (0.49)	0.59 (0.69)	0.72 (0.74)	0.121	0.20 (0.46)	0.22 (0.48)	0.090	0.22 (0.48)	0.56 (0.64)	0.73 (0.75)	**0.026**	0.20 (0.45)	0.21 (0.47)	0.150
Having no one to talk to about issues	0.24 (0.49)	0.32 (0.52)	0.36 (0.57)	0.513	0.23 (0.47)	0.25 (0.50)	0.079	0.36 (0.59)	0.42 (0.63)	0.51 (0.68)	0.260	0.30 (0.54)	0.41 (0.63)	**0.000**
Something bad that happened recently	0.26 (0.57)	0.33 (0.61)	0.37 (0.68)	0.589	0.21 (0.51)	0.30 (0.61)	**0.000**	0.26 (0.57)	0.32 (0.61)	0.32 (0.62)	0.967	0.21 (0.51)	0.30 (0.62)	**0.000**
Thoughts or dreams about bad events^a^	0.23 (0.50)	0.37 (0.64)	0.31 (0.58)	0.333	0.17 (0.44)	0.27 (0.54)	**0.000**	0.21 (0.48)	0.27 (0.50)	0.38 (0.62)	0.088	0.17 (0.44)	0.23 (0.51)	**0.000**

We used t-tests to test for significant differences between the groups. Significant p-values in bold. ^a^ “Thoughts or dreams about bad events from the past, e.g., the destruction of one's own home, physical violence or a sexual act under duress.”

When also considering gender, significant differences were found regarding men and women living at-risk-of-poverty at T1 with women reporting more concerns about weight and looks and more stress at work. Men reported more concerns about low sexual desire. At T2, less affluent women reported more concerns about weight and looks, the burden of caring for children, parents or other family members, and their financial situation. Amongst the more affluent respondents at T1, we found that women reported to be more bothered by almost all psychosocial stressors, except for low sexual desire, financial concerns and having no one to talk to. At the second time point, all stressors were reported as more bothersome by more affluent women compared to more affluent men, with the sole exception of financial concerns. Looking at the differences between the time points, we observed that both genders of the less affluent groups reported increases in concern about weight and looks as well as having no one to talk to, with less affluent men also reporting an increase in lower sexual desire compared to the previous time point. For more affluent men we found significant increases for concern abought weight and looks, sexual desire, problems with their partner and not having anyone to talk to and significant decreases for care burden. More affluent women reported significant increases in almost all items except care burden, worrying about financial issues and the trauma items.

### Depressiveness, anxiety, and loneliness

Table 4 shows the differences in depressiveness, anxiety, and loneliness between the time points for more and less affluent men and women. We observed significant group differences between less and more affluent respondents for all outcomes at both time points with less affluent respondents reporting significantly higher scores. When tested for changes between the two time points, we found that less affluent respondents reported a significant decrease in depressiveness, no significant change in anxiety, and a significant increase in loneliness. Respondents that were more affluent did not demonstrate any significant changes between the time points for depressiveness and anxiety, but a significant increase in loneliness.

**Table 4:** Depressiveness, anxiety, and loneliness of men and women living and not living at-risk-of-poverty (*N* = 8,100).

	**T1**						**T2**					
	**At-risk-of-poverty**		**Not at-risk-of-poverty**				**At-risk-of-poverty**		**Not at-risk-of-poverty**		
	**(*****N*** = **342)**		**(*****N*** = **7,758)**				**(N** = **342)**		**(*****N*** = **7,758)**		

	**M (SD)**		**M (SD)**		***p***		**M (SD)**		**M (SD)**		***p***	
Depressiveness	5.14 (4.45)		4.31 (3.84)		**0.000**		5.04 (4.77)		4.23 (3.94)		**0.000**	
Anxiety	0.94 (1.25)		0.74 (1.06)		**0.000**		1.01 (1.28)		0.74 (1.10)		**0.000**	
Loneliness	3.91 (2.71)		3.57 (2.43)		**0.012**		4.27 (2.76)		3.92 (2.55)		**0.014**	
	**Respondents at-risk-of-poverty over time**						**Respondents not at-risk-of-poverty over time**					
	**T1**		**T2**				**T1**		**T2**		
	**M (SD)**		**M (SD)**		***p***		**M (SD)**		**M (SD)**		***p***	
Depressiveness	5.14 (4.45)		5.04 (4.77)		**0.007**		4.31 (3.84)		4.23 (3.94)		0.580	
Anxiety	0.94 (1.25)		1.01 (1.28)		0.922		0.74 (1.06)		0.74 (1.10)		0.564	
Loneliness	3.91 (2.71)		4.27 (2.76)		**0.000**		3.57 (2.43)		3.92 (2.55)		**0.008**	
	**Respondents at-risk-of-poverty**						**Respondents not at-risk-of-poverty**					
	**T1**			**T2**			**T1**			**T2**	
	**Men**	**Women**		**Men**	**Women**		**Men**	**Women**		**Men**	**Women**	
	**(*****N*** = **154)**	**(*****N*** = **188)**		**(*****N*** = **154)**	**(*****N*** = **188)**		**(*****N*** = **3,870)**	**(*****N*** = **3,888)**		**(*****N*** = **3,870)**	**(*****N*** = **3,888)**	
	**M (SD)**	**M (SD)**	***p***	**M (SD)**	**M (SD)**	***p***	**M (SD)**	**M (SD)**	***p***	**M (SD)**	**M (SD)**	***p***
Depressiveness	4.47 (4.10)	5.69 (4.66)	**0.012**	4.58 (4.13)	5.42 (5.22)	0.104	3.68 (3.55)	4.94 (4.00)	**0.000**	3.56 (3.70)	4.89 (4.05)	**0.000**
Anxiety	0.88 (1.22)	1.04 (1.28)	0.242	0.94 (1.16)	1.06 (1.38)	0.403	0.57 (0.95)	0.90 (1.13)	**0.000**	0.57 (0.96)	0.91 (1.19)	**0.000**
Loneliness	3.64 (3.86)	4.12 (2.81)	0.103	4.12 (2.67)	4.39 (2.83)	0.368	3.27 (2.30)	3.86 (2.52)	**0.000**	3.60 (2.38)	4.25 (2.66)	**0.000**
	**Respondents at-risk-of-poverty**						**Respondents not at-risk-of-poverty**					
	**T1**	**T2**		**T1**	**T2**		**T1**	**T2**		**T1**	**T2**	
	**M (SD)**	**M (SD)**	***p***	**M (SD)**	**M (SD)**	***p***	**M (SD)**	**M (SD)**	***p***	**M (SD)**	**M (SD)**	***p***
Depressiveness	4.47 (4.10)	4.58 (4.13)	0.682	5.69 (4.66)	5.42 (5.22)	0.306	3.68 (3.55)	3.56 (3.70)	**0.005**	4.94 (4.00)	4.89 (4.05)	0.245
Anxiety	0.88 (1.22)	0.94 (1.16)	0.525	1.04 (1.28)	1.06 (1.38)	0.844	0.57 (0.95)	0.57 (0.96)	0.540	0.90 (1.13)	0.91 (1.19)	0.540
Loneliness	3.64 (3.86)	4.12 (2.67)	**0.027**	4.12 (2.81)	4.39 (2.83)	0.131	3.27 (2.30)	3.60 (2.38)	**0.000**	3.86 (2.52)	4.25 (2.66)	**0.000**

We used t-tests to test for significant differences between the groups. Significant p-values in bold.

When taking the interaction of living-at-risk-of-poverty and gender into account, less affluent women only reported significantly higher scores in depressiveness at T1 than less affluent men, while more affluent women reported significantly higher scores in depressiveness, anxiety, and loneliness at both time points compared to more affluent men. Additionally, only more affluent men underwent a significant decrease in depressiveness between the time points. For loneliness, all groups reported significantly higher scores at the second time point, except for women living at-risk-of-poverty.

## Discussion

In this study, we found that respondents living at-risk-of-poverty were not only more likely to experience negative changes in their income and work situation, but also reported significantly higher scores for psychosocial stress, depressiveness, anxiety, and loneliness. At the beginning of the pandemic, they more often received financial compensation than more affluent individuals. Regardless of income, women were found to be more burdened than men. For less affluent individuals, women reported more financial concerns and burdens of caring for children and significant others than men. For more affluent individuals, women reported more negative economic and employment changes during the pandemic, more concerns about numerous psychosocial stress factors, and higher symptom burden in depressiveness, anxiety, and loneliness than men. These results might imply an increase in wealth and gender inequality, which, in turn, might indicate a decline in social cohesion at the beginning of the pandemic. We also found that, between the time points, both the economic impacts as well as the mental health impacts seemed to have declines, implying an incline of social cohesion.

### Economic impact

We observed that less affluent respondents reported significantly more often a reduced income and less working hours since the start of the pandemic while more affluent respondents either did not have any change in income or had an increase both in income and in working hours. Prior studies had similar findings, with Adams-Prassl et al. (2020) concluding that the reduction of working hours or even job loss was more prevalent amongst temporary workers and low-skilled workers which are generally part of the poorest population group. Martinez-Bravo and Sanz (2021) also reported a large discrepancy between the richest and the poorest quintile: The income of the poorest decreased much more than the income of the richest. Additionally, Findling et al. (2021) found that low- to moderate income households suffered and continue to suffer the most financially under the pandemic. Households who had savings before the pandemic reported to have lost those. This might indicate a widening of the wealth gap. In addition to this, our analysis showed that less affluent individuals got more likely financial support only at the beginning of the pandemic and were more likely to start a new job at the four-month follow-up. This might be due to loss of income in the current employment and the wish or need to work full-time without reduced working hours or income. The same was true for more affluent women reporting more often to have started a new job at T2. Probably, they were also unsatisfied with their current work situation as they worked more without increases in income. When testing for significant differences between the time points, we also found a decrease in less affluent respondents reporting to work and earn less. We also observed a decrease in more affluent respondents working and earning more. This might indicate a slow closure of the wealth gap to pre-pandemic levels.

Interestingly we found no significant discrepancies in economic impacts between men and women living at-risk-of-poverty. However, amongst more affluent respondents, we found a gender gap with more affluent women working more hours but more affluent men earning more money. These results contradict the findings of previous studies: Women, in general but in particular mothers, were found to either work less than men or to have lost their jobs during the pandemic due to childcare responsibilities, especially during the beginning of the pandemic (Carli, 2020; Alon et al., 2021; Collins et al., 2021; Hipp and Bünning, 2021; Reichelt et al., 2021). A possible explanation for this result might be that the women in our sample were more likely to work in secure occupations that were also more compatible with childcare (e.g., home office), or that they had a social network helping with childcare. The fact that more affluent men more often reported an increase in income might indicate a widening of the gender gap. Other studies found that women had a larger decrease in income than men. They were also reported to recover much slower financially than men, which might be due to care work responsibilities at home (Martinez-Bravo and Sanz, 2021).

### Psychosocial impact

We found that people living at-risk-of-poverty were generally more affected by psychosocial burdens. For less affluent people, financial, social, and traumatic concerns were of particular interest. This result was to be expected as there is growing literature on children growing up in poverty having a higher risk of being exposed to severe stressors and multiple traumatic events such as witnessing violent events, food insecurity, or maternal depression, which are additionally heightened by the dangerous living environments of urban poverty (Kiser et al., 2008; Briggs-Gowan et al., 2010; Collins et al., 2010). The heightened financial concern amongst less affluent respondents might be due to a lack of financial buffers and resources as well as the inability to cut costs in order to save up money in financially stressful times, which were found predominantly among low-income people (Gennetian and Shafir, 2015). Factors associated with urban poverty have been shown to also be associated with higher risk of family dysfunction and impacted interpersonal relationships, which might explain why less affluent respondents reported significantly more to be burdened by social concerns (Collins et al., 2010). Poverty-related stress has been reported to impact interpersonal relationships in the family (Grant et al., 2003; Conger and Donnellan, 2007). Moreover, these social concerns might also be related to the type of jobs less affluent people usually work: People working in supermarkets experienced a whole new type of stress since they were suddenly considered an “essential” worker, which might have left them with a burden of responsibility and societal stress.

Additionally, they were constantly exposed to a heightened risk of infection. Interestingly, less affluent individuals reported more health concerns at T2, suggesting a greater focus on the pandemic and its health effects with a time lag. Only at T1 did less affluent persons report that something bad happened recently which might be due to loss of income or working hours which was not significant at T2 anymore. Studies during the pandemic found that parents and their adolescent children suffered from a significant increase in psychosocial stress, which was even significantly higher amongst mothers, possibly due to care responsibilities and a generally higher vulnerability to stress disorders (Connor et al., 2020; Paschke et al., 2021). This might also explain why, in our study, less affluent women reported more frequently concerns about caring for children, parents, or other family members, as well as about financial issues at T2.

More affluent women reported more concerns than affluent men in almost all psychosocial stress factors, only did they not report financial worries. Interestingly, while all respondents demonstrated an increase in having no one to talk to during the pandemic, only more affluent women reported significantly more to be burdened with having no one to talk to at T2 compared to their male counterparts. This might indicate the impacts of contact reduction due to social distancing and pandemic measures which might have led to more loneliness. Previous studies showed that working women in particular reported significantly more often to be burdened by multiple co-existing strains such as strains within their occupation, strains in caregiving, but also household chore strains (Kramer and Kipnis, 1995) and are more affected by psychiatric morbidity because of caregiving (Covinsky et al., 2003). The COVID-19 pandemic seems to have reinforced these gender roles after the closure of schools and nurseries, which might have led to an increase in stress among women who are trying to incorporate these role traits into their self-identity (Connor et al., 2020). Families had to take care of their children while also continuing to work. This care work, however, was largely the responsibility of women (Power, 2020).

### Depressiveness, anxiety, and loneliness

We found that less affluent people, who were more affected by the abovementioned stressors, were also generally more affected by depressiveness, anxiety, and loneliness at both time points. Even before the outbreak of COVID-19, members of low-income families experienced a wide array of stressors such as crowding, noise, family turmoil, and early childhood separation, which resulted in psychological distress, impacted well-being, a self-regulation deficit, and maladaptive coping strategies (Evans and English, 2002; Grant et al., 2003; Conger and Donnellan, 2007). Additionally, studies performed during the COVID-19 pandemic highlighted a vicious circle of poverty: Stressors associated with poverty, such as food insecurity and limited access to mental health services, were found to be exacerbated by the stress resulting from the COVID-19 pandemic (Gabrielli and Lund, 2020). Also, multiple studies have identified low income to be a major risk factor for poor mental health outcomes during the pandemic (Daly et al., 2020; Peters et al., 2020; Santabárbara et al., 2020; Breslau et al., 2021; Fancourt et al., 2021; Kunzler et al., 2021; Niedzwiedz et al., 2021; Benatov et al., 2022; Bonati et al., 2022; Ernst et al., 2022). Although we found a significant decrease in depressiveness between time points among less affluent respondents, the symptom burden generally remained higher than among more affluent individuals. Additionally, more affluent men showed a significant decrease in depressiveness. Previous longitudinal research, too, has reported slight increases at the beginning of the pandemic and decreases in the course of the pandemic for anxiety and depression symptoms (Peters et al., 2020; Kivi et al., 2021; Kunzler et al., 2021; Prati and Mancini, 2021). Therefore, it is unsurprising that we found decreases in depressiveness.

We also observed significant increases in loneliness for both less and more affluent respondents over time. This might be associated with the significant increase in all respondents reporting to not having anyone to talk to. When additionally testing for gender differences, we found that all groups with the exception of women living at-risk-of-poverty reported significantly increased levels of loneliness over time. Previous research showed that loneliness was an important health factor that increased significantly during the pandemic, especially among females and people of low income (Bu et al., 2020; Varga et al., 2021; Jaspal and Breakwell, 2022). The insignificant increase in our study for less affluent women might be due to this group's low number of cases.

### Implications for social cohesion

When we put these results into the framework of social cohesion, we suggest that the widening of the wealth gap and the gender gap indicate a decline in social cohesion (Kawachi and Kennedy, 1997). Additionally, as Wilkinson and Pickett (2010) argued, due to the rises in inequality, a person's status becomes an increasingly important factor of one's identity, which in turn increases status competition, social evaluation, and status anxiety. People further down the social ladder become more disadvantaged in regards to this status competition since they gathered fewer material and immaterial resources such as high income, good jobs, houses, cars, as well as social connections, which might increase their social standing. To prove this theory, the author's presented evidence from WHO data that linked anxiety to inequality. As mentioned, we were able to find a rise in inequality as well as significantly higher symptom burdens amongst less affluent respondents, which appears to confirm Wilkinson and Pickett's results.

Between the two time points, however, we found a significant decrease in less affluent respondents that stated to working less and having a reduced income. This might indicate the beginning of a decrease in the wealth gap to pre-pandemic levels and imply that social cohesion also increased back to pre-pandemic levels, while inequality decreased. To add to that, while we found initially heightened scores for depression and anxiety, we found either no significant changes or even a decline over the course of our study. This might indicate that, because social cohesion possesses a protective quality for mental health, it might have increased between the two time points (Friedkin, 2004; Fone et al., 2007; Greene et al., 2015; Borkowska and Laurence, 2021). This corroborates the findings by Silveira et al. (2022) as well as Borkowska and Laurence (2021) who found that the levels of social cohesion declined during lockdown (end of 2020), but increased after governmental measures were lifted (beginning of 2021).

Because of these results, we suggest a further reduction of the income disparities between less and more affluent people by the means government issued financial aid as well as a strengthening of social cohesion in deprived neighborhoods in order to address mental health impacts following the pandemic.

### Limitations

The most important limitation is the small number of cases per group, so the effects described are probably rather small. The 4.2% proportion of people living at-risk-of-poverty within our sample is an underrepresentation of the actual percentage amongst the German population [18.7% in 2018, (Statista, 2022)]. Though is must be noted that the Federal Statistical Office took respondents of all ages into account while our sample was only compromised of individuals aged 25 to 88. Nevertheless, those respondents within our sample that can be categorized as living at-risk-of-poverty match the characteristics found within the German population: they were mostly younger people (aged 18–24, in our sample 25–34), people living alone, working part-time, irregularly or were unemployed, as well as people with a low to moderate level of education, with a migration background and individuals who were single parents that live on the threshold of poverty (Statistisches Bundesamt, 2021). Additionally, the two survey time points might have been too close in time to one another, which might have influenced some results and rendered some otherwise significant factors insignificant. Finally, though a large body of research suggested that poverty and inequality in general have an impact on social cohesion, the direction of the causality might also be the other way around. A low social cohesion might increase inequality due to lack of trust, mutual tolerance, and discrimination, which can manifest itself in the absence of or discrimination in the distribution of governmental aid, such as welfare and subvention programs. Consequently, more longitudinal research needs to be done concerning the causal association between social cohesion, mental health, and poverty.

## Data availability statement

The datasets presented in this article are not readily available because the datasets presented in this article are not allowed to be publicly shared according to regulations for data protection (EU General Data Protection Regulations). The data used during the current study are exclusively available at the local database. Requests to access the datasets should be directed to PW, Philipp.Wild@unimedizin-mainz.de.

## Ethics statement

The studies involving human participants were reviewed and approved by the Ethics Committee of the Rhineland-Palatinate Medical Association as well as the Data Protection Officer of the Johannes Gutenberg University Hospital Mainz. The patients/participants provided their written informed consent to participate in this study.

## Author contributions

JP: idea, statistical analysis, and manuscript. NH: manuscript and critical feedback. RB, PW, NP, TM, JK, KL, and MB: study design and critical feedback. All authors contributed to the article and approved the submitted version.

## Funding

The Gutenberg COVID Study (GCS) was funded by the European Regional Development Fund (ERDF/REACT-EU), the Ministry of Science and Health of Rhineland-Palatinate, the ReALity Initiative of the Life Sciences of Johannes Gutenberg University Mainz, and the National Research Network of University Medicine (NUM).

## Conflict of interest

This paper is part of the author JP's cumulative PhD. The remaining authors declare that the research was conducted in the absence of any commercial or financial relationships that could be construed as a potential conflict of interest.

## Publisher's note

All claims expressed in this article are solely those of the authors and do not necessarily represent those of their affiliated organizations, or those of the publisher, the editors and the reviewers. Any product that may be evaluated in this article, or claim that may be made by its manufacturer, is not guaranteed or endorsed by the publisher.
