# Supplementary material for: The burdens of poverty during the COVID-19 pandemic
Source: Front Sociol. 2022 Nov 24;7:995318. doi: 10.3389/fsoc.2022.995318 (PMC9731111; doi:10.3389/fsoc.2022.995318)
Supplement: Supplementary file 1 [file Data_Sheet_1.PDF]

Supplementary Material

Appendix 1. Changes in income and employment during the COVID-19 pandemic for men and women living and not living at-risk-of-poverty over time (N = *8100*).

At-risk-of-poverty over time						
	At-risk-of-poverty (*N = 342*)			Not at-risk-of-poverty (*N = 7758*)		
	T1	T2	p	T1	T2	p
Change in personal income during pandemic						
No	233 (68,7%)	228 (73,6%)	**0,026**	5731 (75,0%)	5907 (80,7%)	**0,000**
Yes, more	19 (5,6%)	15 (4,8%)	0,670	912 (11,9%)	663 (9,0%)	**0,000**
Yes, less	78 (23,0%)	50 (16,1%)	**0,000**	876 (11,5%)	551 (7,5%)	**0,000**
Change in working hours/occupation						
No	102 (29,8%)	131 (38,3%)	**0,007**	1762 (22,7%)	3353 (43,2%)	**0,000**
Yes, working less	35 (10,2%)	14 (4,1%)	**0,000**	518 (6,7%)	135 (1,7%)	**0,000**
Yes, working more	17 (5,0%)	19 (5,6%)	0,684	631 (8,1%)	430 (5,6%)	**0,000**
Yes, I got a new job	4 (1,2%)	8 (2,3%)	0,206	96 (1,2%)	72 (0,9%)	0,056
Yes, I lost my job	3 (0,9%)	2 (0,6%)	NA	20 (0,3%)	8 (0,1%)	NA
Yes, I received short-time compensation	11 (3,2%)	1 (0,3%)	NA	112 (1,4%)	57 (0,7%)	NA
Yes, I received financial aid	3 (0,9%)	1 (0,3%)	0,318	14 (0,2%)	7 (0,1%)	0,127

At-risk-of-poverty x gender over time												
	At-risk-of-poverty (*N = 342*)						Not at-risk-of-poverty (*N = 7758*)					
	Men (*N = 154*)			Women (*N = 188*)			Men (*N = 3870*)			Women (*N = 3888*)		
	T1	*T2*		*T1*	*T2*		*T1*	*T2*		*T1*	*T2*	
	N (%)	N (%)	*p*	N (%)	N (%)	*p*	N (%)	N (%)	*p*	N (%)	N (%)	*p*
Change in personal income during pandemic												
No	106 (68,8%)	106 (76,3%)	**0,033**	127 (68,7%)	122 (71,3%)	0,305	2859 (74,8%)	2914 (79,6%)	**0,000**	2872 (75,2%)	2993 (81,7%)	**0,000**
Yes, more	10 (6,5%)	7 (5,0%)	0,368	9 (4,9%)	8 (4,7%)	0,764	476 (12,5%)	392 (10,7%)	**0,002**	436 (11,4%)	271 (7,4%)	**0,000**
Yes, less	35 (22,7%)	21 (15,1%)	**0,011**	43 (23,2%)	29 (17,0%)	**0,009**	441 (11,5%)	278 (7,6%)	**0,000**	435 (11,4%)	273 (7,5%)	**0,000**
Change in working hours/occupation												
No	54 (35,1%)	65 (42,2%)	0,146	48 (25,5%)	66 (35,1%)	**0,018**	927 (24,0%)	1738 (44,9%)	**0,000**	835 (21,5%)	1615 (41,5%)	**0,000**
Yes, working less	16 (10,4%)	6 (3,9%)	**0,018**	19 (10,1%)	8 (4,3%)	**0,007**	263 (6,8%)	61 (1,6%)	**0,000**	255 (6,6%)	74 (1,9%)	**0,000**
Yes, working more	4 (2,6%)	6 (3,9%)	0,481	13 (6,9%)	13 (6,9%)	1,000	268 (6,9%)	173 (4,5%)	**0,000**	363 (9,3%)	257 (6,6%)	**0,000**
Yes, I got a new job	1 (0,6%)	3 (2,0%)	0,319	3 (1,6%)	5 (2,7%)	0,416	39 (1,0%)	26 (0,7%)	0,096	57 (1,5%)	46 (1,2%)	0,264
Yes, I lost my job	1 (0,6%)	1 (0,7%)	NA	2 (1,1%)	1 (0,5%)	NA	9 (0,2%)	3 (0,0%)	NA	11 (0,3%)	5 (0,1%)	NA
Yes, I received short-time compensation	4 (2,6%)	0 (0,0%)	NA	7 (3,7%)	1 (0,5%)	NA	59 (1,5%)	30 (0,8%)	NA	53 (1,4%)	27 (0,7%)	NA
Yes, I received financial aid	0 (0,0%)	1 (0,7%)	0,319	3 (1,6%)	0 (0,0%)	0,083	5 (0,1%)	4 (0,1%)	0,739	9 (0,2%)	3 (0,1%)	0,083

Note: We used chi-square tests of independence to test for significant differences between the time points. Significant p-values in bold

Appendix 2. Stressors and burdens of men and women living and not living at-risk-of-poverty over time (*N = 8100*).

At-risk-of-poverty over time						
	**At-risk-of-poverty** (*N = 342*)			**Not at-risk-of-poverty** (*N = 7758*)		
	**T1**	**T2**		**T1**	**T2**	
	M (SD)	M (SD)	***p***	M (SD)	M (SD)	***p***
Sum score PHQ stress	4,66 (3,47)	4,96 (3,58)	0,084	3,98 (3,15)	4,20 (3,33)	**0,000**
Concern about health	0,76 (0,70)	0,83 (0,68)	0,147	0,71 (0,65)	0,73 (0,67)	**0,007**
Concern about weight and looks	0,66 (0,67)	0,77 (0,71)	**0,000**	0,59 (0,67)	0,70 (0,69)	**0,000**
Low or no sexual desire or pleasure during intercourse	0,42 (0,62)	0,53 (0,66)	**0,013**	0,50 (0,65)	0,55 (0,67)	**0,000**
Problems with spouse or (life) partner	0,44 (0,65)	0,44 (0,60)	0,933	0,38 (0,59)	0,41 (0,61)	**0,000**
Burden of caring for children, parents or other family members	0,46 (0,65)	0,46 (0,67)	0,308	0,45 (0,66)	0,43 (0,65)	**0,001**
Stress at work or in school	0,54 (0,72)	0,54 (0,72)	0,550	0,59 (0,73)	0,58 (0,72)	0,247
Financial issues or concerns	0,66 (0,72)	0,65 (0,71)	0,853	0,21 (0,47)	0,20 (0,46)	0,432
Having no one to talk to about issues	0,34 (0,55)	0,47 (0,66)	**0,000**	0,24 (0,48)	0,36 (0,59)	**0,000**
Something bad that happened recently	0,36 (0,65)	0,32 (0,61)	0,545	0,26 (0,56)	0,26 (0,57)	0,885
Thoughts or dreams about bad events[a]	0,34 (0,61)	0,33 (0,57)	0,713	0,22 (0,50)	0,20 (0,48)	**0,000**

At-risk-of-poverty x gender over time												
	**At-risk-of-poverty** (*N = 342*)						**Not at-risk-of-poverty** (*N = 7758*)					
	**Men** (*N = 154*)			**Women** (*N = 188*)			**Men** (*N = 3870*)			**Women** (*N = 3888*)		
	**T1**	**T2**		**T1**	**T2**		**T1**	**T2**		**T1**	**T2**	
	M (SD)	M (SD)	***p***	M (SD)	M (SD)	***p***	M (SD)	M (SD)	***p***	M (SD)	M (SD)	***p***
Sum score PHQ stress	4,27 (3,54)	4,58 (3,47)	0,235	4,98 (3,40)	5,27 (3,64)	0,210	3,59 (2,96)	3,70 (3,17)	**0,010**	4,37 (3,29)	4,69 (3,40)	**0,000**
Concern about health	0,71 (0,69)	0,81 (0,70)	0,139	0,80 (0,71)	0,84 (0,66)	0,509	0,65 (0,63)	0,66 (0,65)	0,337	0,77 (0,66)	0,80 (0,68)	**0,004**
Concern about weight and looks	0,51 (0,63)	0,64 (0,69)	**0,004**	0,79 (0,68)	0,87 (0,71)	**0,027**	0,49 (0,62)	0,57 (0,64)	**0,000**	0,70 (0,70)	0,83 (0,71)	**0,000**
Low or no sexual desire or pleasure during intercourse	0,50 (0,64)	0,56 (0,66)	0,182	0,35 (0,59)	0,49 (0,66)	**0,035**	0,49 (0,64)	0,53 (0,66)	**0,000**	0,51 (0,66)	0,57 (0,68)	**0,000**
Problems with spouse or (life) partner	0,44 (0,65)	0,40 (0,58)	0,619	0,44 (0,65)	0,48 (0,62)	0,565	0,36 (0,57)	0,38 (0,59)	**0,007**	0,40 (0,61)	0,44 (0,63)	**0,000**
Burden of caring for children, parents or other family members	0,40 (0,65)	0,37 (0,61)	0,889	0,51 (0,65)	0,55 (0,71)	0,180	0,39 (0,61)	0,36 (0,60)	**0,005**	0,52 (0,70)	0,50 (0,70)	0,062
Stress at work or in school	0,42 (0,65)	0,47 (0,69)	0,077	0,62 (0,76)	0,59 (0,75)	0,416	0,54 (0,69)	0,52 (0,69)	0,180	0,65 (0,76)	0,65 (0,75)	**0,727**
Financial issues or concerns	0,59 (0,69)	0,56 (0,64)	0,278	0,72 (0,74)	0,73 (0,75)	0,448	0,20 (0,46)	0,20 (0,45)	0,839	0,22 (0,48)	0,21 (0,47)	0,373
Having no one to talk to about issues	0,32 (0,52)	0,42 (0,63)	**0,009**	0,36 (0,57)	0,51 (0,68)	**0,002**	0,23 (0,47)	0,30 (0,54)	**0,000**	0,25 (0,50)	0,41 (0,63)	**0,000**
Something bad that happened recently	0,33 (0,61)	0,32 (0,61)	0,730	0,37 (0,68)	0,32 (0,62)	0,617	0,21 (0,51)	0,21 (0,51)	0,386	0,30 (0,61)	0,30 (0,62)	0,585
Thoughts or dreams about bad events[a]	0,37 (0,64)	0,27 (0,50)	0,091	0,31 (0,58)	0,38 (0,62)	0,242	0,17 (0,44)	0,17 (0,44)	0,516	0,27 (0,54)	0,23 (0,51)	**0,000**

Note: We used chi-square tests of independence to test for significant differences between the time points. Significant p-values in bold. [a] "Thoughts or dreams about bad events from the past, e.g., the destruction of one's own home, physical violence or a sexual act under duress.
